# Access of medication review for people living with dementia: An analysis of inequality using Swedish register data

**DOI:** 10.1002/alz.70909

**Published:** 2025-11-13

**Authors:** Sanjib Saha, Sofie Persson, Johan Jarl, Dominic Trepel, Cecilia Lenander, Elisabet Londos, Patrik Midlöv, Brian Lawlor, Håkan Toresson, UlfG Gerdtham

**Affiliations:** ^1^ Department of Clinical Science (Malmö) Health Economics Unit Lund University Lund Sweden; ^2^ Trinity Institute of Neurosciences (TCIN), Trinity College Dublin Ireland; ^3^ The Swedish Institute for Health Economics (IHE) Lund Sweden; ^4^ Department of Clinical Sciences in Malmö Centre for Primary Health Care Research Lund University Malmö Sweden; ^5^ Department of Clinical Science (Malmö), Clinical Memory Research Unit Lund University Malmö Sweden; ^6^ Centre for Economic Demography Lund University Lund Sweden; ^7^ Department of Economics Lund University Lund Sweden

**Keywords:** medication management, medication review, pharmaceutical review, registry data

## Abstract

**INTRODUCTION:**

This study examines medication reviews (MRs) among people living with dementia (PWD) in Sweden, focusing on frequency and factors influencing access.

**METHODS:**

Using nationwide register data, all diagnosed PWD from 2010 to 2016 were assessed for MR receipt in 2015 and/or 2016. The analysis guided by the Andersen healthcare utilization framework, considered predisposing, enabling, and need‐based factors through multiple logistic regression.

**RESULTS:**

The MR rate was 16% in 2015, 21% in 2016, and 30% across both years. Increased MR likelihood was linked to higher comorbidity (adjusted odds ratio [aOR] = 1.45, *p* = 0.00), polypharmacy (aOR = 1.55, *p* = 0.00), and residential care (aOR = 3.92, *p* = 0.00), while being married or cohabiting reduced (aOR = 0.80, *p* = 0.00) MR receipt.

**DISCUSSION:**

Despite national guidelines recommending annual MRs since 2012, rates remain low, especially outside residential care. The findings highlight the need for more equitable MR access for all PWD, particularly those not in residential care.

**Highlights:**

- Only 16%–21% of people with dementia received a medication review in 2015–2016 in Sweden.
- Higher comorbidity, polypharmacy, and residential care increased likelihood of receiving a medication review.
- Being married or cohabiting lowered the chance of receiving a medication review.
- Education level and gender did not affect access to medication reviews.

## 1 INTRODUCTION

Medication management for people living with dementia (PWD) is particularly challenging due to the complexity of the condition and its associated comorbidities. As the prevalence of dementia continues to rise globally, appropriate medication use in this vulnerable population has become increasingly critical.^1^ PWD frequently have multiple comorbidities, necessitating various medications, putting them at high risk of polypharmacy, typically defined as the concurrent use of five or more medications, and potentially inappropriate medications (PIMs) use.^1^ Polypharmacy in PWD is associated with an increased risk of adverse events, drug interactions, medication nonadherence, and prolonged hospitalizations.^2^ PIM use is particularly concerning as it is associated with higher hospitalization rates and increased risk of falls, cardiovascular event, and mortality.^3^, ^4^ Moreover, cognitive impairment in dementia significantly impacts medication adherence and increases the risk of medication errors, leading to an increased dependence on caregivers for medication management.^1^

Medication reviews (MRs) have emerged as a strategy to address these concerns,^5^ recommended in the Swedish national guidelines.^6^ An MR is a structured assessment of patients medications, aiming to improve the safety and quality in pharmaceutical treatment.^7^ These reviews are particularly crucial for PWD, as they help identify PIMs, assess the benefits and risks of prescribed drugs, and adjust treatment goals as the disease progresses.^8^ MR is a collaborative approach that involves the PWD, informal caregivers, and healthcare professionals in decision‐making, ensuring that the treatment aligns with individual needs and preferences. Pharmacist‐led or multidisciplinary team‐based medication evaluations have been shown to reduce both the average number of medications per patient and the prevalence of PIM use.^8^ Sharma et al. reported a reduction in the average number of PIMs from 1.5 to 0.9 per patient within 180 days of an MR.^5^ Despite these benefits, implementing MRs for PWD remains a challenge.^9^

Swedish law states that there should be equal access to healthcare for equal need irrespective of factors like disease type, age, gender, ethnicity, or economic status. In Sweden, the National Board of Health and Welfare (NBHW) introduced national recommendations for MRs in 2012 to reduce inappropriate prescribing and prevent medication‐related problems.^6^ NBHW recommends that all patients aged 75 years or older who are prescribed five or more medications should receive an MR when in contact with outpatient care, inpatient care, starting homecare, or moving to a residential care setting. A review report also required at the discharge from the hospital if the patient received an MR at inpatient care. However, in Sweden's decentralized healthcare system each healthcare region are independently accountable for operationalizing and implementing these suggestions, which leads to variations in MR provision. In Region Skåne, MR eligibility criteria are more specific than the national guidelines and were implemented before the NBHW recommendations. Patients qualify for an MR if they meet at least one of the following conditions^10^: (1) residing in residential care (annual MR); (2) aged 65 years or older and receiving municipality‐provided home care (annual MR); (3) aged 75 years or older, prescribed five or more medications and hospitalized; (4) deemed in need of an MR by their treating physician.

Consequently, the majority of PWD in Region Skåne are anticipated to meet the criteria for yearly MR. However, the actual proportion of PWD receiving an MR and the factors influencing access to these reviews remain unknown. Therefore, this study aims to investigate the frequency of MR in Region Skåne, Sweden, and identify demographic, clinical, and medication‐related factors associated with MR access.

**RESEARCH IN CONTEXT**

1. **Systematic review**: We conducted a systematic review of prior literature using national and international databases to identify studies on medication review (MR) access among people living with dementia (PWD). Previous research highlights the benefits of MRs in reducing polypharmacy and inappropriate medication use in PWD but also reveals significant variability in MR implementation and limited evidence on factors influencing MR access.
2. **Interpretation**: Our study, using comprehensive Swedish register data, demonstrates that MR rates among PWD remain low despite national guidelines, with clinical need (comorbidity, polypharmacy, and residential care) rather than socioeconomic factors driving access. These findings align with prior reports of underutilization and inequity in MR provision, especially for community‐dwelling PWD.
3. **Future directions**: Future research is needed to clarify whether low MR rates reflect under‐registration or limited implementation, explore barriers to MR access among community‐dwelling PWD, and evaluate the impact of MRs on quality of life, mortality, and healthcare resources utilization.

## 2 METHODS

### 2.1 Data

Sweden has a long history of national registers compiled by government agencies and other organizations that are available for research purposes and the Swedish Personal Identification Number enables linkage between registers. For this study, we created a database of all PWD in Region Skåne who received a first dementia diagnosis between 2010 and 2016, as identified in Region Skåne's Healthcare Database using ICD‐10 codes F00.0–F00.2, F00.9, G30.0, G30.1, G30.8, and G30.9 for AD diagnoses, as well as F01.1–F01.3, F01.8–F02.0, F02.2–F02.4, F02.8, F03.9, F03–P, F10.6–7A, G32.0, and G31.8 for other types of dementia. Information on MR was obtained from the same database according to the Swedish procedure coding system (Classification of Health Care Measures, KVÅ). There are two types of MRs: (1) simple MR (KVÅ XV015) conducted by the treating physician, and (2) in‐depth MR (KVÅ XV016) typically provided by a multidisciplinary team together with the patient (details provided in the Supplement). We define PWD receiving an MR if they receive either simple MR or in‐depth MR. However, in a sensitivity analysis, we consider only in‐depth MR, since in‐depth MR is associated with a financial benefit to the providers.

Following the introduction of the NBHW recommendations for MRs in 2012, the codes for MR have been accessible in Swedish healthcare registers since 2013, such as the National Patient Register (NPR). However, as the NPR only covers inpatient and specialized outpatient care, it lacks information on MRs conducted in primary healthcare centers. We therefore limit our investigation to the Region Skåne, where the Region Skåne's Healthcare Database includes data from the primary care in addition to specialized outpatient and inpatient care. For this study, we assessed whether a PWD received an MR in 2015 and 2016 separately, as well as both years pooled.

The study was approved by the Regional Research Ethics Board at Lund University (dnr 2017/554)

### 2.2 Conceptual model

**FIGURE 1:**
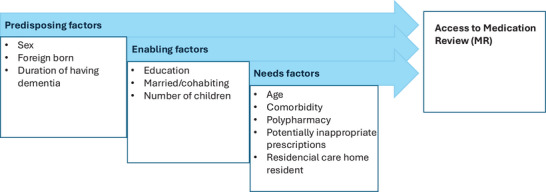
Theoretical framework based on Andersen healthcare utilization model.

The Andersen Healthcare utilization framework was employed to identify factors influencing MR access^11^ to facilitating the interpretation of results and makes policy recommendations. The model categorizes variables into three groups: (1) Predisposing factors: characteristics that influence an individual's inclination and capacity to manage health decline, including prompt health care‐seeking behavior. (2) Enabling factors: resources that facilitate access to healthcare, such as knowledge of where to seek care. (3) Need factors: the individual's health care needs, which can be perceived by the PWD or family members or evaluated by health care professionals. Figure 1 presents the model alongside the variables analyzed in this study.

### 2.3 Variables

The primary outcome is a binary variable of whether a PWD received an MR. Variables associated with access to MR are included based on prior research of healthcare access.^12^, ^13^ Following the Andersen model, the variables included in predisposing factors are sex, foreign background (born outside Sweden or having both parents born outside Sweden vs. born in Sweden with at least one Swedish‐born parent), and duration of dementia (< 2 years, 2–4 years, and 5–6 years). Variables included in the enabling factors are highest attained educational level, marital status (married or cohabitant vs. single), and number of children. The need factors have current age (categorized as < 75 years, 75–84 years, and ≥ 85 years, based on Region Skåne's MR eligibility guidelines), comorbidity measured with the Elixhauser Comorbidity Index (ECI) (0 = no comorbidity, 1–2 = some comorbidity, > 2 = high comorbidity), and living in residential care.

Medication‐related variables, such as polypharmacy and potentially inappropriate prescription (PIP), were based on dispensation data from the Swedish Prescribed Drug Register^14^ using Anatomical Therapeutic Chemical (ATC) codes. Polypharmacy was defined as having ≥ 5 medications within a single month in a specific year. Drugs that the NBHW recommends should be avoided for PWD^15^ was termed as PIP in line with previous research.^16^, ^17^ These include concurrent use of three or more psychotropic drugs (from any of the groups hypnotics‐sedatives, antipsychotics, anxiolytics, and antidepressants) (PIP I), drugs with anticholinergic properties (urinary and gastrointestinal antispasmodics, anticholinergic antiemetics, class Ia antiarrhythmics, anticholinergic antiparkinsonian drugs, low‐potency antipsychotics, tricyclic antidepressants, and first‐generation antihistamines) (PIP II) and long‐acting benzodiazepines (diazepam, nitrazepam, or flunitrazepam) (PIP III). The three criteria were analyzed separately.[Fig alz70909-fig-0002]

### 2.4 Statistical analysis

The chi‐squared test was used to test differences in proportion of receiving an MR. Both univariable and multivariable logistic regression models was used to estimate the unadjusted and adjusted odds ratios (aORs) with 95% confidence intervals (CIs) to investigate what factors were associated with access to MR.

Post‐estimation tests were performed to ensure optimal model performance of the logistic regressions. Goodness of fit was assessed using the Hosmer–Lemeshow goodness‐of‐fit test. Multicollinearity was checked with a variance inflation factor (VIF) threshold of < 10. Statistical significance was defined as *p* < 0.05. All analyses were performed using STATA 18.^18^

### 2.5 Scenario and sensitivity analyses

We performed scenario analyses to explore access to MR among PWD groups that, according to the recommendations in Region Skåne, should have annual access to MR. The following four groups are identified based on these recommendations:

1. Persons living in a residential care home;
2. Persons aged above 74 years and exposed to polypharmacy;
3. Persons 65 years of age and receiving municipality‐provided home care;
4. Persons experiencing polypharmacy and meeting the three PIP criteria outlined above, as a proxy for being deemed in need of an MR by a treating physician.

All scenario analyses were adjusted for the variables in the main analysis. In sensitivity analysis, the fully adjusted model was re‐run for PWD who received in‐depth MRs only.

## 3 RESULTS

**FIGURE 2 alz70909-fig-0002:**
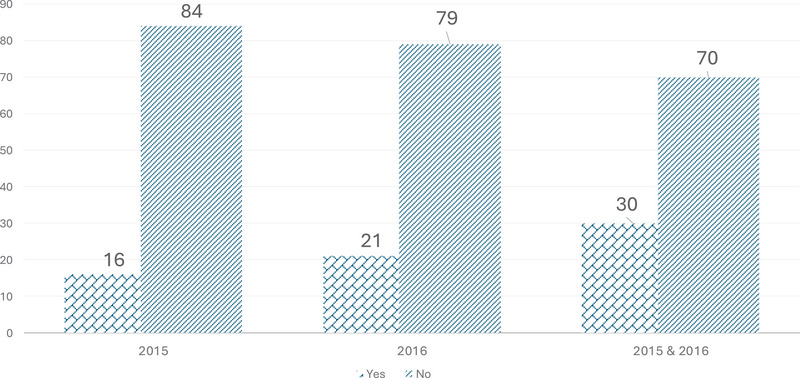
Proportion of persons living with dementia who received medication review according to study year.

The proportion of PWD who had a registered MR was 16% in 2015 and 21% in 2016 (Figure 2), while 30% of PWD received an MR within the 2‐year period. The likelihood of receiving an MR varied significantly across several predisposing, enabling, and need factors (Table 1), with the number of children being the only enabling factor that was not statistically significant.

**TABLE 1:** Characteristics of the study population who received medication review.

	Medication review		
Characteristics	No (%)	Yes (%)	*p*‐value
**Predisposing factors**			
Sex			0.00
Women	4213 (67)	2038 (33)	
Men	3099 (74)	1096 (26)	
Foreign background			0.00
No	6143 (69)	2706 (31)	
Yes	1169 (73)	426 (27)	
Duration of dementia			0.00
< 2 years	2532 (76)	788 (24)	
2 to 3 years	2409 (68)	1128 (32)	
4 and above	2371 (66)	1218 (34)	
**Enabling factors**			
Education			0.00
Compulsory	3294 (68)	1540 (32)	
Upper secondary	2488 (70)	1071 (30)	
Higher secondary	1317 (74)	471 (26)	
Missing	213 (80)	52 (20)	
Married/cohabiting			0.00
No	4586 (65)	2451 (35)	
Yes	2726 (80)	683 (20)	
Children			0.646
None	1133 (70)	475 (30)	
1−2	4008 (70)	1749 (30)	
3 and above	2171 (70)	910 (30)	
**Needs factors**			
Age			0.00
Below 75	1565 (78)	433 (22)	
75 to 84	2787 (72)	1074 (28)	
85 and higher	2960 (65)	1627 (35)	
Comorbidity (Elixhauser index)			0.00
Zero	927 (80)	237 (20)	
1 to 2	3069 (73)	1112 (27)	
3 and above	3316 (65)	1785 (35)	
Poly pharmacy			0.000
No	1889 (82)	409 (18)	
Yes	5423 (67)	2725 (33)	
Living in residential care			0.000
No	5042 (84)	972 (16)	
Yes	2270 (51)	2162 (49)	
PIP I			0.000
No	6621 (72)	2570 (28)	
Yes	691 (55)	564 (45)	
PIP II			0.000
No	6427 (71)	2659 (29)	
Yes	885 (65)	475 (35)	
PIP III			0.000
No	4961 (76)	1582 (24)	
Yes	2351 (60)	1582 (40)	

*Note*: *p*‐Value is based on chi‐square test.

In Table 2, we present unadjusted and adjusted odds ratios for receiving an MR in year 2015–2016. Several characteristics remained significantly associated with MR receipt in the fully adjusted model (Table 2). Among the predisposing factors, having dementia for 2–3 years (aOR 1.28, 95% CI 1.14−1.44, *p* = 0.000) or 4+ years (aOR 1.25, 95% CI 1.11−1.40, *p* = 0.00) increased the likelihood of receiving an MR compared to a duration of < 2 years. Among enabling factors, being married or cohabiting was negatively associated with receiving an MR (aOR 0.80, 95% CI 0.71−0.89, *p* = 0.000). Among the need factors, a high comorbidity (aOR 1.45, 95% CI 1.21−1.72, *p* = 0.000), polypharmacy (aOR 1.55, 95% CI 1.36−1.78, *p* = 0.000), and all the three PIP were associated with a greater likelihood of receiving an MR. Finally, PWD living in residential care were substantially more likely to receive an MR (aOR 3.92, 95% CI 3.53−4.34, *p* = 0.000) than PWD living in community setting. In the sensitivity analyses for “in depth” MR, PIP I and PIP II, which were significantly associated with an increased likelihood of MR in the base case model, were insignificant (Table S1).

**TABLE 2:** Factors affecting access to medication review in Region Skåne.

Characteristics	Unadjusted odds ratio (95% CI)	*p*‐value	Adjusted odds ratio (95% CI)	*p*‐value
**Predisposing factors**				
Sex				
Women	1.00	−	1.00	−
Men	0.73 (0.67−0.79)	0.000	0.96 (0.87−1.06)	0.41
Foreign background				
No	1.00	−	1.00	−
Yes	0.83 (0.74−0.94)	0.000	0.93 (0.82−1.06)	0.30
Duration of dementia				
<2 years	1.00	−	1.00	−
2 to 3 years	1.50 (1.35−1.67)	0.000	1.28 (1.14−1.44)	0.000
4 and above	1.65 (1.48−1.83)	0.000	1.25 (1.11−1.4)	0.000
**Enabling factors**				
Education				
Compulsory	1.00	−	1.00	−
Upper secondary	0.92 (0.83−1.01)	0.08	1.06 (0.96−1.18)	0.256
Higher secondary	0.76 (0.68−0.86)	0.00	1.02 (0.90−1.17)	0.730
Missing	0.52 (0.38−0.71)	0.00	0.67 (0.48−0.95)	0.023
Married/cohabiting				
No	1.00	−	1.00	−
Yes	0.47 (0.43−0.52)	0.00	0.80 (0.71−0.89)	0.00
Children				
None	1.00	−	1.00	−
1−2	1.04 (0.92−1.17)	0.52	0.99 (0.87−1.14)	0.922
3 and above	1.0 (0.58−1.14)	0.10	0.99 (0.86−1.15)	0.905
**Needs factors**				
Age				
Below 75	1.00	−	1.00	−
75 to 84	1.39 (1.22−1.58)	0.00	1.1 (0.96−1.33)	0.186
85 and higher	1.99 (1.75‐2.24)	0.00	1.16 (1.0−1.34)	0.036
Comorbidity (Elixhauser index)				
Zero	1.00	−	1.00	−
1 to 2	1.42 (1.21−1.66)	0.00	1.12 (0.94−1.33)	0.209
3 and above	2.11 (1.81−2.45)	0.00	1.45 (1.21−1.72)	0.00
Polypharmacy				
No	1.00	−	1.00	−
Yes	2.32 (2.06−2.61)	0.00	1.55 (1.36−1.78)	0.00
Living in residential care				
No	1.00	−	1.00	−
Yes	4.94 (4.5−5.4)	0.00	3.92 (3.53−4.34)	0.00
PIP I				
No	1.00	−	1.00	
Yes	2.10 (1.9−2.4)	0.00	1.24 (1.07−1.43)	0.03
PIP II				
No	1.00	−	1.00	
Yes	1.30 (1.1−1.5)	0.00	1.22 (1.07−1.40)	0.03
PIP III				
No	1.00	−	1.00	
Yes	2.07 (1.9−2.6)	0.00	1.13 (1.02−1.26)	0.018

**FIGURE 3 alz70909-fig-0003:**
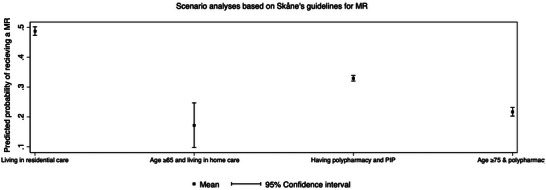
Predicted probability of receiving a medication review in four scenarios.

Results from the scenario analyses revealed that PWD who met the Region Skåne's eligibility criteria for MRs had a higher probability of receiving an MR compared to the overall average across all PWD. The highest probability was observed among PWD living in residential care homes at 49% (Figure 3).

## 4 DISCUSSION

Using Swedish register data, we found that the rates of registered MR among PWD were low in 2015–2016. This aligns with findings from a recent audit by the Health and Social Care Inspectorate (IVO) of Sweden, which reported that half of the residents in retirement homes lacked documented MRs.^19^ However, it is possible that MRs are not consistently registered and thus unavailable in healthcare registers, which would indicate that the rate reported in the current study is a lower bound. Discussions with pharmacists involved in conducted MRs in Region Skåne suggest that especially simple MR (XV015) are inconsistently registered. Our data confirm/show that fewer cases of simple MR are registered compared to in‐depth MR, although we cannot establish within the current study if this is reflecting a true difference in performed MR or differences in registration patterns. Although we have no reason to suspect that there is a systematic difference in what MR are registered, this cannot be ruled out and could potentially bias the analysis on associated factors. However, the sensitivity analyses on in‐depth MR separately, which can be expected to have a higher rate of registration and therefore less potential for systematic differences, are largely concordant with the main results. This supports that the estimation of associations is unbiased.

### 4.1 Predisposing factors

In the univariable analysis, women were more likely to receive an MR than men, but this association disappeared after adjusting for other factors (Table 2). Given the well‐documented influence of sex and gender on health inequities,^20^ this finding is particularly relevant in dementia care. Women account for two‐thirds of dementia cases due to longer life expectancy, disease differences, and sex‐ and gender‐related risk factors. Older women often have lower educational attainment, less physical activity, and a higher risk of depression, a major dementia risk factor, compared to men.^21^ A scoping review found inadequate understanding of both sex and gender differences in drug use among older adults with dementia, indicating a need for more research in this area.^22^ It is therefore encouraging that we found no inequality in terms of sex in access to MR in the Swedish healthcare system. We likewise found no differences based on immigration status.

The likelihood of accessing MR was found to be higher with longer duration of dementia. This is expected, as treatment goals may shift, requiring regular medication adjustments, as the disease progresses. Cognitive decline increases the risk of medication mismanagement, making periodic reassessment crucial.^23^ Furthermore, deprescribing PIM becomes more relevant in advanced stages of dementia.^24^

### 4.2 Enabling factors

Education level did not influence MR receipt, despite higher education often has been shown to be associated with better healthcare access.^25^ While education is associated with dementia risk,^26^ it has limited impact on medication effectiveness or appropriateness post diagnosis. Instead, education plays a more significant role for caregivers and healthcare providers in understanding the disease and improving communication.

Conversely, being married or cohabiting was negatively associated with access to MR. One possible explanation is that this may be due to the caregiving dynamics in the spousal relationship. Spouses often take on primary caregiving responsibilities, which can create significant physical and emotional burdens.^27^ Overwhelmed spouses may lack the capacity to recognize the need for MRs or to advocate for medication changes.^1^, ^28^ Alternatively, spouses may act as “watchdogs” by successfully taking on the responsibility of monitoring and reporting medication problems, thereby ensuring medication adherence and directly communicating with physicians to modify prescriptions, bypassing formal MR documentation.^29^ Spouses are commonly involved in decision‐making for medication changes, as well as medication management.^29^, ^30^ Consequently, spouses are likely to observe alterations in the condition of PWD attributable to a specific prescription and may interact with the treating physician to modify the medication without necessitating an MR or registration in the system. Further research, particularly of a qualitative kind, is necessary to comprehend this mechanism.

Finally, the number of children did not influence likelihood of MR, indicating that caregiving support depends more on availability and capacity rather than family size.^31^, [Fig alz70909-fig-0003]

### 4.3 Need factors

All included need‐based factors were significantly associated with MR receipt. Higher comorbidity levels increased the probability of MR, as managing multiple chronic conditions often necessitates complex medication regimens, heightening the risk of drug‐related problems, including adverse drug reactions and PIP.^5^, ^25^, ^32^ PWD are particularly vulnerable to polypharmacy and PIP‐related risks^4^, ^5^ further highlighting the need for regular MR. However, while all three PIP measurements were positively associated with MR in the base case analysis, only use of benzodiazepine (PIP III) were associated with in‐depth MR (Table S1). One possible reason is the perceived need of PIP for PWDs as PIP III had the higher prevalence in our study comparing to other PIPs (Table 1). Benzodiazepine is also used for insomnia in addition to anxiety and agitation for PWD,^33^ which may come to the treating physician as a request from the caregivers to reduce the caregiving burden. This indicates variations in the indicators of MR, which should be analyzed in future studies.

Living in residential care was the factor strongest associated with MR receipt, even after adjusting for all other variables. This is likely due to the structured healthcare environment and the presence of interdisciplinary teams. Residential care facilities often have established protocols for regular MR to manage polypharmacy and ensure the medication appropriateness, as well as trained personnel that can pick up on medication side effects. Studies have shown that up to 83% of residential care residents in Norway receive MR, leading to significant reductions of PIM use.^5^, ^34^ The collaborative approach in residential care, involving pharmacists, nurses, general practitioners, and specialists, facilitates comprehensive medication reviews. In contrast, community‐dwelling PWD may lack consistent healthcare access and structured medication monitoring systems.

Overall, these results indicate that access to MR is primarily driven by clinical need and disease progression rather than enabling factors such as education, socioeconomic status, and family structure.

### 4.4 Scenario analyses

Among PWDs who clearly met the criteria for MR, the number receiving MR was substantially higher than the average, although still relatively low (Figure 3). For those living in residential care, 49% obtained an MR, that is, 51% did not have access to MR. This may represent a practitioner effect, where MRs were routinely conducted at specific centers or accurately registered. The probability of receiving an MR increased with older age, polypharmacy and PIP, and consistent with existing recommendations.

### 4.5 Limitation

Our analysis identifies associations only, preventing any conclusions based on causality for the factors influencing MR. Furthermore, key clinical variables, such as dementia severity and specific dementia types, were unavailable. Drug–drug interactions, a particular category of PIP, could not be assessed due to data constraints. Information on over‐the‐counter medication use was also lacking, as this is not registered in the Prescribed Drug Register. Furthermore, the dataset did not capture healthcare organizational factors, such as clinician training, pharmacists’ involvement, and dementia‐specific management guidelines.^35^, ^36^ It should be noted that the data is somewhat old. However, as this is the first explorative study using real‐world, population‐based data to identify characteristics associated with MR among PWD, it makes an important contribution to the field.

## 5 FUTURE RESEARCH AND POLICY IMPLICATIONS

It is important to recognize that the present study highlights, rather than resolve, key barriers to access and implementation of MR in PWD. Future research should investigate if the low rates of MR are attributable to inadequate registration or due to low implementation. Although particular care is necessary to resolve either of these issues, the latter is likely being more urgent. Moreover, exploratory research is necessary to identify why cohabiting was, contrary to expectations, negatively associated with MR. The relationship between PIP and in‐depth MR warrants further study as well. Additionally, future studies should examine the impact of MR on quality of life, mortality, and healthcare resource utilization to investigate the overall benefits of appropriate medication management. Such efforts could inform decision makers on the importance of MR for PWD. While this study does not provide immediate solutions, the insights gained can inform the development of targeted strategies, such as enhancing inter‐professional collaboration, increasing patient and caregiver engagement in shared decision‐making, and improving healthcare system supports—that are essential for overcoming these barriers.

## 6 CONCLUSION

The rates of registered MRs for PWD are low in Region Skåne, Sweden. People with dementia with polypharmacy, potentially inappropriate prescriptions, multiple comorbidities, and those living in residential care are more likely to receive a medication review. However, based on our scenario analyses, maximum 50% of eligible persons are gaining access in any given year.

## CONFLICT OF INTEREST STATEMENT

Håkan Toresson is an owner of Cell Invent AB as well as a board member in Cell Invent AB and Vivobakt AB. Johan Jarl have received consulting fees from Ramboll. Dominic Trépel has received funding from the Global Brain Health Institute. Sanjib Saha, Sofie Persson, Cecilia Lenander, Elisabet Londos, Patrik Midlöv, Brian Lawlor, and Ulf‐G. Gerdtham have nothing to disclose. Author disclosures are available in the Supporting Information.

## CONSENT STATEMENT

This is register‐based research; therefore, consent was not necessary. The study was approved by the Regional Research Ethics Board at Lund University (dnr 2017/554) and was performed in accordance with the ethical standards laid down by the 1964 Declaration of Helsinki and its later amendments.

## Supporting information



Supporting Information

Supporting Information
