# Supplementary material for: Access of medication review for people living with dementia: An analysis of inequality using Swedish register data
Source: Alzheimers Dement. 2025 Nov 13;21(11):e70909. doi: 10.1002/alz.70909 (PMC12614082; doi:10.1002/alz.70909)
Supplement: Supplementary file 1 — Supporting Information [file ALZ-21-e70909-s002.docx]

Supplementary Table 1: Factors affecting access to “in-depth” medication review

| Characteristics | Adjusted Odds ratio (95% CI) | p value |
| --- | --- | --- |
| *Predisposing factors* | | |
| Sex |  |  |
| Women® | 1.00 | − |
| Men | 0.92 (0.82−1.03) | 0.150 |
| Foreign background |  |  |
| No® | 1.00 | − |
| Yes | 0.94 (0.81−1.09) | 0.434 |
| Duration of dementia |  |  |
| Less than 2 years® | 1.00 | − |
| 2 to 3 years | 1.40 (1.23−1.60) | **0.000** |
| 4 and above | 1.42 (1.24−1.61) | **0.000** |
| *Enabling factors* | | |
| Education |  |  |
| Compulsory® | 1.00 | − |
| Upper secondary | 1.05 (0.94−1.18) | 0.406 |
| Higher secondary | 0.96 (0.82−1.11) | 0.565 |
| Missing | 0.77 (0.53−1.12) | 0.177 |
| Married/cohabiting |  |  |
| No® | 1.00 | − |
| Yes | 0.69 (0.60−0.78) | **0.000** |
| Children |  |  |
| None® | 1.00 | − |
| 1-2 | 0.92 (0.79−1.07) | 0.281 |
| 3 and above | 0.96 (0.81−1.12) | 0.579 |
| *Needs Factors* | | |
| Age |  |  |
| Below 75® | 1.00 | − |
| 75 to 84 | 1.30 (1.10−1.55) | 0.002 |
| 85 and higher | 1.54 (1.30−1.82) | 0.000 |
| Comorbidity (Elixhauser index) |  |  |
| Zero® | 1.00 | − |
| 1 to 2 | 1.23 (1.0−1.50) | **0.047** |
| 3 and above | 1.59 (1.29−1.94) | **0.000** |
| Poly pharmacy |  |  |
| No® | 1.00 | − |
| Yes | 1.86 (1.59−2.18) | **0.00** |
| Living in residential care |  |  |
| No® | 1.00 | − |
| Yes | 6.18 (2.74−3.51) | **0.00** |
| PIP I |  |  |
| No® | 1.00 | − |
| Yes | 1.09 (0.9−1.27) | 0.268 |
| PIP II |  |  |
| No® | 1.00 | − |
| Yes | 1.09 (0.9−1.28) | 0.227 |
| PIP III |  |  |
| No® | 1.00 | − |
| Yes | 1.14 (1.02−1.28) | **0.022** |

Note: Bold means statistically significant

**Definitions of Medication Reviews in Sweden**

Two types of MRs are provided in Sweden, 1) Simple (“Enkel”) and 2) In-depth (“Fördjupad”).

1) Simple (“Enkel”) MR: According to NBHW, Swedish care providers shall offer patients who are 75 years or older and who are prescribed at least five drugs a simple MR when the patient 1) visits a doctor in outpatient care, 2) enrolls in inpatient care, 3) starts home care, or 4) moves to a nursing home. The simple MR is generally provided by a doctor where the intention is to map as far as possible 1) which drugs the patient are prescribed and why, 2) which of these drugs the patient uses, and 3) which other drugs the patient uses. The doctor must check whether the drug list is correct and assess whether the drug treatment is appropriate and safe. The simple MR must be provided to the eligible person once a year. However, a patient and his/her caregivers can also request a simple MR even though they are not eligible for a review.

2) In-depth (“Fördjupad”) MR: The patient who after a simple MR has persistent drug-related problems or where there is a suspicion of such problems, should be is offered a comprehensive in-depth drug review by the care providers. In an “in-depth” MR, for each prescribed drug, the providers needs to 1) check that there is an indication for the drug, 2) evaluate the treatment effect, 3) assess how the dosage of the drug relates to the patient’s physiological functions, 4) evaluate the drug's side effects, the risk of side effects, and the risk of interactions being greater than the benefit of the drug, and 5) evaluate the benefit of the drug in relation to the patient's other drugs and treatments. In the “in-depth” MR, it is also important to know any other kind of drug use such as herbal, supplements and over the counter medication. The patient must receive individually tailored information about the results of the review and the review should be followed up. A decision must be made as to when the follow-up is to be done, and which care provider or care unit is to be responsible for it. The in-depth drug review must be designed and carried out in consultation with the patient who is part of the team. However, in many situations, it is not possible for the doctor to decide and assess all aspects of an “in-depth” review, therefore, most often, the “in-depth” review is conducted by a team. Depending on how complex the treatment is and what resources are available and what needs the patient has, the team may look different. The team may include nurse, pharmacists, physiotherapists, occupational therapists, dietitians, and other care providers. This also varies from region to region, for example, Skåne region requires multi professional to perform “in-depth” MR.
